# Supplementary material for: Clinical and molecular characterization of patients fulfilling Chompret criteria for Li-Fraumeni syndrome in Southern Brazil
Source: PLoS One. 2021 Sep 16;16(9):e0251639. doi: 10.1371/journal.pone.0251639 (PMC8445435; doi:10.1371/journal.pone.0251639)
Supplement: S1 Fig — (DOCX) [file pone.0251639.s001.docx]

Unrelated patients with suggestive phenotype of LFS

(n=211)

Public Hospital

(n=148)

Patients from the Cancer Genetics Clinic

(n=108)

Retrospective analysis of Sanger and MLPA results

(n=68)

NGS + MLPA analysis

(n=38)

MGPT including TP53 variants (NGS) and rearrangements

(n=2)

Patients from the Pediatric Cancer Ward

(n=40)

Retrospective analysis of Sanger and MLPA results

(n=29)

NGS + MLPA analysis

(n=11)

Private Clinics (Hospital Moinhos de Vento, Clinionco, Hospital Mãe de Deus and Instituto de Oncologia Kaplan)

(n=43)

Retrospective analysis of Sanger and MLPA results

(n=3)

MGPT including TP53 variants (NGS) and rearrangements

(n=40)

Excluded for not meeting Chrompret criteria after review or living outside Southern Brazil

(n=20)

**S1 Fig. Consort Diagram representing the patient recruitment and genetic testing process employed in the current study.**
