# Supplementary material for: Clinical and molecular characterization of patients fulfilling Chompret criteria for Li-Fraumeni syndrome in Southern Brazil
Source: PLoS One. 2021 Sep 16;16(9):e0251639. doi: 10.1371/journal.pone.0251639 (PMC8445435; doi:10.1371/journal.pone.0251639)
Supplement: S1 Table — (DOCX) [file pone.0251639.s002.docx]

**S1 Table. 2015 Revised Chompret criteria for LFS and *TP53* gene testing.**

| **2015 version of Chompret criteria**  **(probands must meet at least one criterion)** | |
| --- | --- |
| **Familial presentation** | Proband with a tumor belonging to LFS tumor spectrum (eg, premenopausal breast cancer, soft tissue sarcoma, osteosarcoma, CNS tumor, adrenocortical carcinoma) before age 46 years, AND at least one first- or second-degree relative with LFS tumor (except breast cancer if proband has breast cancer) before age 56 years or with multiple tumors |
| **Multiple primitive tumors** | Proband with multiple tumors (except multiple breast tumors), two of which belong to LFS tumor spectrum and first of which occurred before age 46 years |
| **Rare tumors** | Patient with adrenocortical carcinoma, choroid plexus tumor, or rhabdomyosarcoma of embryonal anaplastic subtype, irrespective of age at diagnosis or family history |
| **Early onset breast cancer** | Breast cancer before age 31 years |

CNS, Central Nervous System; LFS, Li-Fraumeni Syndrome.
