# Supplementary material for: Clinical and molecular characterization of patients fulfilling Chompret criteria for Li-Fraumeni syndrome in Southern Brazil
Source: PLoS One. 2021 Sep 16;16(9):e0251639. doi: 10.1371/journal.pone.0251639 (PMC8445435; doi:10.1371/journal.pone.0251639)
Supplement: S2 Table — (DOCX) [file pone.0251639.s003.docx]

**S2 Table.** **Clinical and molecular characterization of all probands (n=191) included in the study.**

| **Proband ID / Gender** | **Age at 1^st^ cancer diagnosis (years)** | **Proband’s type of cancer** | **Age at diagnosis, other tumors (years)** | **2015 Version Chompret Criterion(s)** | **Recruitment** | **Genetic Testing** | **chr17 position on Assembly GRCh37 (dbSNP rs ID)** | ***TP53* variant HGVS c.** | ***TP53* variant HGVS p.** |
| --- | --- | --- | --- | --- | --- | --- | --- | --- | --- |
| 1 / M | 84 | Lung | NA | Familial | PUB | NGS + MLPA | WT | WT | WT |
| 2 / F | 44 | Breast | NA | Familial | PUB | Sanger + MLPA | WT | WT | WT |
| 3 / F | 33 | Breast | NA | Familial | PUB | Sanger + MLPA | WT | WT | WT |
| 4 / M | 8 | Leukemia | NA | Familial | PED | Sanger + MLPA | WT | WT | WT |
| 5 / M | 16 | Leukemia | NA | Familial | PED | Sanger + MLPA | WT | WT | WT |
| 6 / F | 40 | Breast | NA | Familial | PUB | Sanger + MLPA | WT | WT | WT |
| 7 / F | 26 | Breast | NA | EOBC | PC | Sanger + MLPA | WT | WT | WT |
| 8 / F | 30 | Breast | NA | EOBC | PC | Sanger + MLPA | WT | WT | WT |
| 9 / F | 29 | Breast | NA | EOBC | PUB | Sanger + MLPA | WT | WT | WT |
| 10 / F | 30 | Breast | NA | EOBC | PC | MGPT | WT | WT | WT |
| 11 / F | 37 | Ovarian sarcoma | NA | Familial | PUB | Sanger + MLPA | WT | WT | WT |
| 12 / F | 36 | Papillary Thyroid | NA | Familial | PC | MGPT | WT | WT | WT |
| 13 / F | 30 | Breast | NA | EOBC | PUB | Sanger + MLPA | WT | WT | WT |
| 14 / M | 9 | Sarcoma | NA | Familial | PED | Sanger + MLPA | WT | WT | WT |
| 15 / F | 27 | Breast | NA | EOBC | PC | MGPT | WT | WT | WT |
| 16 / F | 7 | Leukemia | NA | Familial | PED | NGS + MLPA | WT | WT | WT |
| 17 / F | 3 | Wilms | NA | Familial | PED | NGS + MLPA | WT | WT | WT |
| 18 / F | 43 | Breast | NA | Familial | PUB | Sanger + MLPA | WT | WT | WT |
| 19 / F | 34 | Breast | Breast (44) | Familial | PUB | Sanger + MLPA | WT | WT | WT |
| 20 / M | 3 | Leukemia | NA | Familial | PED | NGS + MLPA | WT | WT | WT |
| 21 / F | 3 | Leukemia | Breast (26) | Familial | PC | MGPT | WT | WT | WT |
| 22 / F | 36 | Breast | NA | Familial | PC | MGPT | WT | WT | WT |
| 23 / F | 32 | Breast | NA | Familial | PUB | NGS + MLPA | WT | WT | WT |
| 24 / F | 49 | Colon | NA | Familial | PUB | Sanger + MLPA | WT | WT | WT |
| 25 / F | 42 | Breast | NA | Familial | PUB | NGS + MLPA | WT | WT | WT |
| 26 / F | 8 | Leukemia | NA | Familial | PED | NGS + MLPA | WT | WT | WT |
| 27 / F | 30 | Breast | NA | EOBC | PC | MGPT | WT | WT | WT |
| 28 / M | 2 | Leukemia | NA | Familial | PUB | NGS + MLPA | WT | WT | WT |
| 29 / F | 30 | Breast | NA | EOBC | PUB | Sanger + MLPA | WT | WT | WT |
| 30 / F | 34 | Breast | NA | Familial | PUB | Sanger + MLPA | WT | WT | WT |
| 31 / F | 36 | Breast | NA | Familial | PUB | NGS + MLPA | WT | WT | WT |
| 32 / F | 42 | Breast | Breast (bilateral) (59) | Familial | PC | MGPT | WT | WT | WT |
| 33 / F | 43 | Breast | NA | Familial | PUB | NGS + MLPA | WT | WT | WT |
| 34 / F | 29 | Breast | Thyroid (27) | EOBC | PC | MGPT | WT | WT | WT |
| 35 / F | 43 | Breast | NA | Familial | PUB | Sanger + MLPA | WT | WT | WT |
| 36 / F | 33 | Breast | NA | Familial | PUB | Sanger + MLPA | WT | WT | WT |
| 37 / F | 25 | Breast | NA | EOBC | PC | MGPT | WT | WT | WT |
| 38 / F | 29 | Breast | Breast (47) | EOBC | PUB | Sanger + MLPA | WT | WT | WT |
| 39 / F | 4 | Breast | NA | Familial | PED | Sanger + MLPA | WT | WT | WT |
| 40 / M | 5 | Rhabdomyosarcoma | NA | Familial | PED | NGS + MLPA | WT | WT | WT |
| 41 / F | 41 | Breast | Gastric (60) | Familial | PUB | Sanger + MLPA | WT | WT | WT |
| 42 / M | 15 | OS | NA | Familial | PED | NGS + MLPA | WT | WT | WT |
| 43 / F | 30 | Breast | NA | EOBC | PC | MGPT | WT | WT | WT |
| 44 / F | 27 | Breast | Breast (30) | EOBC | PC | MGPT | WT | WT | WT |
| 45 / F | 11 | Abdominal sarcoma | NA | Familial | PED | Sanger + MLPA | WT | WT | WT |
| 46 / F | 50 | Breast | NA | Familial | PUB | Sanger + MLPA | WT | WT | WT |
| 47 / F | 28 | Breast | NA | EOBC | PUB | Sanger + MLPA | WT | WT | WT |
| 48 / F | 23 | Breast | Parotid (47) | EOBC | PUB | Sanger + MLPA | WT | WT | WT |
| 49 / M | 6 | Leukemia | NA | Familial | PUB | NGS + MLPA | WT | WT | WT |
| 50 / F | 48 | Breast (bilateral) | NA | Familial | PUB | NGS + MLPA | WT | WT | WT |
| 51 / F | 32 | OS | NA | Familial | PUB | NGS + MLPA | WT | WT | WT |
| 52 / F | 27 | Breast | NA | EOBC | PC | MGPT | WT | WT | WT |
| 53 / F | 34 | Breast | NA | Familial | PC | MGPT | WT | WT | WT |
| 54 / F | 29 | Breast | NA | EOBC | PC | MGPT | WT | WT | WT |
| 55 / F | 28 | Breast | NA | EOBC | PUB | Sanger + MLPA | WT | WT | WT |
| 56 / M | 17 | OS | NA | Familial | PUB | NGS + MLPA | WT | WT | WT |
| 57 / M | 9 | CNS | NA | Familial | PED | Sanger + MLPA | WT | WT | WT |
| 58 / F | 26 | Breast | NA | EOBC | PUB | Sanger + MLPA | WT | WT | WT |
| 59 / F | 29 | Breast | NA | EOBC | PC | MGPT | WT | WT | WT |
| 60 / F | 28 | Breast | NA | EOBC | PUB | Sanger + MLPA | WT | WT | WT |
| 61 / F | 28 | Breast | NA | EOBC | PC | MGPT | WT | WT | WT |
| 62 / F | 28 | Breast | NA | EOBC | PUB | Sanger + MLPA | WT | WT | WT |
| 63 / F | 4 | ACC | NA | RT | PED | Sanger + MLPA | WT | WT | WT |
| 64 / F | 49 | Breast | NA | Familial | PUB | NGS + MLPA | WT | WT | WT |
| 65 / F | 25 | Breast | NA | EOBC | PUB | NGS + MLPA | WT | WT | WT |
| 66 / F | 29 | Breast | Breast (34) | EOBC | PUB | Sanger + MLPA | WT | WT | WT |
| 67 / F | 39 | Breast | NA | Familial | PC | MGPT | WT | WT | WT |
| 68 / F | 24 | Breast | NA | EOBC | PUB | MGPT | WT | WT | WT |
| 69 / M | 4 | Ewing | NA | Familial | PED | NGS + MLPA | WT | WT | WT |
| 70 / M | 10 | OS | NA | Familial | PED | Sanger + MLPA | WT | WT | WT |
| 71 / F | 26 | Breast | NA | Familial, EOBC | PC | MGPT | WT | WT | WT |
| 72 / M | 59 | ACC | NA | RT | PUB | Sanger + MLPA | WT | WT | WT |
| 73 / F | 39 | Breast | Uterus (39) | Familial | PUB | NGS + MLPA | WT | WT | WT |
| 74 / F | 4 | Wilms | NA | Familial | PUB | NGS + MLPA | WT | WT | WT |
| 75 / F | 23 | Breast + Breast sarcoma | Breast sarcoma (45) | EOBC | PUB | Sanger + MLPA | WT | WT | WT |
| 76 / M | 10 | CNS | NA | Familial | PUB | Sanger + MLPA | WT | WT | WT |
| 77 / F | 23 | ACC | NA | RT | PUB | Sanger + MLPA | WT | WT | WT |
| 78 / M | 5 | Ewing | NA | Familial | PED | Sanger + MLPA | WT | WT | WT |
| 79 / F | 26 | Breast | NA | EOBC | PUB | Sanger + MLPA | WT | WT | WT |
| 80 / F | 44 | Breast | NA | Familial | PC | MGPT | WT | WT | WT |
| 81 / F | 29 | Breast | NA | EOBC | PC | MGPT | WT | WT | WT |
| 82 / F | 28 | Breast | NA | Familial | PUB | Sanger + MLPA | WT | WT | WT |
| 83 / F | 30 | Breast | NA | EOBC | PC | MGPT | WT | WT | WT |
| 84 / F | 41 | Breast | NA | Familial | PUB | NGS + MLPA | WT | WT | WT |
| 85 / M | 2 | Leukemia | NA | Familial | PED | Sanger + MLPA | WT | WT | WT |
| 86 / F | 10 | CNS | NA | Familial | PED | NGS + MLPA | WT | WT | WT |
| 87 / F | 5 | Neroblastoma | NA | Familial | PUB | NGS + MLPA | WT | WT | WT |
| 88 / F | 3 | PNET | NA | Familial | PED | NGS + MLPA | WT | WT | WT |
| 89 / F | 26 | Breast | NA | EOBC | PUB | Sanger + MLPA | WT | WT | WT |
| 90 / F | 37 | Breast | NA | Familial | PUB | NGS + MLPA | WT | WT | WT |
| 91 / F | 64 | Breast | NA | Familial | PUB | Sanger + MLPA | WT | WT | WT |
| 92 / F | 44 | Breast | Melanoma (52) | Familial | PC | MGPT | WT | WT | WT |
| 93 / F | 28 | Breast | NA | EOBC | PC | MGPT | WT | WT | WT |
| 94 / F | 29 | Breast | NA | EOBC | PUB | NGS + MLPA | WT | WT | WT |
| 95 / F | 38 | Breast | Breast (59) | Familial | PUB | NGS + MLPA | WT | WT | WT |
| 96 / F | 30 | Breast | NA | EOBC | PUB | Sanger + MLPA | WT | WT | WT |
| 97 / F | 7 | Rhabdomyosarcoma | NA | Familial | PED | Sanger + MLPA | WT | WT | WT |
| 98 / M | 14 | OS | NA | Familial | PED | Sanger + MLPA | WT | WT | WT |
| 99 / M | 47 | GIST | Kidney (55) | Familial | PUB | Sanger + MLPA | WT | WT | WT |
| 100 / F | 34 | Breast | NA | Familial | PC | MGPT | WT | WT | WT |
| 101 / F | 30 | Breast | NA | EOBC | PC | MGPT | WT | WT | WT |
| 102 / F | 49 | Breast | NA | Familial | PUB | NGS + MLPA | WT | WT | WT |
| 103 / F | 48 | Breast | NA | Familial | PUB | NGS + MLPA | WT | WT | WT |
| 104 / F | 16 | Ewing | NA | Familial | PED | Sanger + MLPA | WT | WT | WT |
| 105 / M | 8 | CNS | NA | Familial | PED | Sanger + MLPA | WT | WT | WT |
| 106 / F | 26 | Breast | NA | EOBC | PUB | Sanger + MLPA | WT | WT | WT |
| 107 / F | 28 | Breast | NA | EOBC | PUB | Sanger + MLPA | WT | WT | WT |
| 108 / F | 28 | Breast | NA | EOBC | PUB | Sanger + MLPA | WT | WT | WT |
| 109 / F | 51 | Breast | NA | Familial | PUB | Sanger + MLPA | WT | WT | WT |
| 110 / F | 62 | Ovary | NA | Familial | PUB | NGS + MLPA | WT | WT | WT |
| 111 / F | 44 | Breast | NA | Familial | PC | MGPT | WT | WT | WT |
| 112 / F | 26 | Breast (bilateral) | NA | EOBC | PUB | NGS + MLPA | WT | WT | WT |
| 113 / F | 45 | Breast | NA | Familial | PUB | Sanger + MLPA | WT | WT | WT |
| 114 / F | 39 | Breast | NA | Familial | PUB | MGPT | WT | WT | WT |
| 115 / F | 45 | Breast | NA | Familial | PUB | Sanger + MLPA | WT | WT | WT |
| 116 / F | 3 | Leukemia | NA | Familial | PED | Sanger + MLPA | WT | WT | WT |
| 117 / F | 29 | Breast | NA | EOBC | PC | MGPT | WT | WT | WT |
| 118 / F | 50 | Breast | NA | Familial | PUB | Sanger + MLPA | WT | WT | WT |
| 119 / F | 27 | Breast | Breast (31) | EOBC | PUB | NGS + MLPA | WT | WT | WT |
| 120 / F | 40 | Breast | NA | Familial | PC | MGPT | WT | WT | WT |
| 121 / F | 50 | Breast | NA | Familial | PUB | NGS + MLPA | WT | WT | WT |
| 122 / F | 23 | Breast | NA | EOBC | PC | MGPT | WT | WT | WT |
| 123 / F | 30 | Breast | NA | EOBC | PUB | NGS + MLPA | WT | WT | WT |
| 124 / F | 36 | Breast | NA | Familial | PUB | Sanger + MLPA | WT | WT | WT |
| 125 / F | 29 | Breast | NA | EOBC | PC | MGPT | WT | WT | WT |
| 126 / F | 29 | Breast | NA | EOBC | PC | MGPT | WT | WT | WT |
| 127 / F | 51 | Colon | Breast (51) | Familial | PUB | Sanger + MLPA | WT | WT | WT |
| 128 / F | 48 | Breast | Endometrium (49) | Familial | PUB | Sanger + MLPA | WT | WT | WT |
| 129 / F | 30 | Breast | NA | EOBC | PC | MGPT | WT | WT | WT |
| 130 / F | 42 | Breast | Breast (55) | Familial | PUB | Sanger + MLPA | WT | WT | WT |
| 131 / M | 4 | Neuroblastoma | NA | Familial | PED | Sanger + MLPA | WT | WT | WT |
| 132 / F | 27 | Breast | NA | EOBC | PC | MGPT | WT | WT | WT |
| 133 / F | 42 | Breast | NA | Familial | PC | MGPT | WT | WT | WT |
| 134 / F | 30 | Breast | NA | EOBC | PUB | NGS + MLPA | WT | WT | WT |
| 135 / F | 27 | Breast | NA | EOBC | PUB | Sanger + MLPA | WT | WT | WT |
| 136 / F | 28 | Breast | NA | EOBC | PUB | Sanger + MLPA | WT | WT | WT |
| 137 / F | 27 | Breast | NA | EOBC | PUB | Sanger + MLPA | WT | WT | WT |
| 138 / F | 28 | Breast | NA | EOBC | PUB | Sanger + MLPA | WT | WT | WT |
| 139 / M | 10 | OS | NA | Familial | PED | NGS + MLPA | WT | WT | WT |
| 140 / F | 9 | Ewing | NA | Familial | PED | Sanger + MLPA | WT | WT | WT |
| 141 / M | 1 | CNS | NA | Familial | PED | NGS + MLPA | WT | WT | WT |
| 142 / F | 39 | Breast | NA | Familial | PC | MGPT | WT | WT | WT |
| 143 / F | 45 | Breast | NA | Familial | PUB | NGS + MLPA | WT | WT | WT |
| 144 / F | 35 | Breast | NA | Familial | PUB | Sanger + MLPA | WT | WT | WT |
| 145 / F | 55 | Breast | NA | Familial | PUB | Sanger + MLPA | WT | WT | WT |
| 146 / F | 12 | OS | Breast (34) | Familial | PC | MGPT | WT | WT | WT |
| 147 / F | 24 | Breast | NA | EOBC | PUB | NGS + MLPA | WT | WT | WT |
| 148 / F | 30 | Breast | NA | EOBC | PC | MGPT | WT | WT | WT |
| 149 / F | 9 | Thryroid | Breast (43) | Familial | PUB | NGS + MLPA | WT | WT | WT |
| 150 / F | 40 | Breast | NA | Familial | PUB | NGS + MLPA | WT | WT | WT |
| 151 / F | 27 | Breast | Breast (44) | EOBC | PUB | Sanger + MLPA | WT | WT | WT |
| 152 / F | 35 | Breast | NA | Familial | PUB | Sanger + MLPA | WT | WT | WT |
| 153 / F | 23 | Breast | NA | EOBC | PUB | NGS + MLPA | WT | WT | WT |
| 154 / F | 44 | Breast | NA | Familial | PUB | Sanger + MLPA | WT | WT | WT |
| 155 / F | 43 | Breast (bilateral) | NA | Familial | PUB | Sanger + MLPA | WT | WT | WT |
| 156 / F | 30 | Breast | NA | Familial, EOBC | PUB | NGS + MLPA | WT | WT | WT |
| 157 / M | 8 | Leukemia | NA | Familial | PED | Sanger + MLPA | WT | WT | WT |
| 158 / M | 46 | Non-Hodgkin’s lymphoma | NA | Familial | PUB | NGS + MLPA | WT | WT | WT |
| 159 / F | 24 | Breast | NA | EOBC | PUB | Sanger + MLPA | WT | WT | WT |
| 160 / F | 25 | CNS | Breast (48) | Familial | PUB | Sanger + MLPA | WT | WT | WT |
| 161 / F | 42 | Breast | NA | Familial | PUB | Sanger + MLPA | WT | WT | WT |
| 162 / F | 26 | Breast | Breast (46) | EOBC | PUB | Sanger + MLPA | WT | WT | WT |
| 163 / F | 41 | Breast | NA | Familial | PUB | NGS + MLPA | WT | WT | WT |
| 164 / M | 44 | Sarcoma | NA | Familial | PUB | NGS + MLPA | WT | WT | WT |
| 165 / M | 0 | ACC | NA | RT | PUB | Sanger + MLPA | WT | WT | WT |
| 166 / F | 32 | Breast | Breast (38) | Familial | PC | Sanger + MLPA | rs28934874 | c. 451C>T | p.( Pro151Ser) |
| 167 / F | 30 | Breast (bilateral) | Thyroid (37) | Familial, EOBC | PUB | Sanger + MLPA | rs1057517983 | c.731G>A | p.( Gly244Asp) |
| 168 / F | 11 | CNS | NA | Familial | PUB | Sanger + MLPA | rs28934575 | c.733G>A | p.( Gly245Ser) |
| 169 / F | 12 | OS | Breast (21), Breast (22), STS(24) | MT, EOBC | PC | MGPT | rs28934575 | c.733G>A | p.( Gly245Ser) |
| 170 / F | 25 | Breast | NA | EOBC | PC | MGPT | rs11540652 | c.743G>A | p.( Arg248Gln) |
| 171 / M | 44 | ACC | NA | RT | PUB | NGS + MLPA | rs121912652 | c.772G>A | p.( Glu258Lys) |
| 172 / F | 19 | OS | Breast (29), STS (38) | Familial, MT, EOBT | PUB | Sanger + MLPA | rs28934576 | c.818G>A | p.( Arg273His) |
| 173 / F | 5 | CNS (CPC) | NA | RT | PC | MGPT | rs28934574 | c.844C>T | p.( Arg282Trp) |
| 174 / F | 0 (6 mo) | ACC | NA | RT | PED | Sanger + MLPA | rs121912664 | c.1010G>A | p.( Arg337His) |
| 175 / F | 0 (4 mo) | ACC | NA | Familial, RT | PED | Sanger + MLPA | rs121912664 | c.1010G>A | p.( Arg337His) |
| 176 / F | 0 (8 mo) | ACC | NA | RT | PED | Sanger + MLPA | rs121912664 | c.1010G>A | p.( Arg337His) hz |
| 177 / F | 1 | ACC | NA | Familial, RT | PUB | Sanger + MLPA | rs121912664 | c.1010G>A | p.( Arg337His) |
| 178 / M | 1 | ACC | NA | RT | PED | Sanger + MLPA | rs121912664 | c.1010G>A | p.( Arg337His) |
| 179 / M | 2 | ACC | NA | RT | PED | Sanger + MLPA | rs121912664 | c.1010G>A | p.( Arg337His) |
| 180 / M | 2 | ACC | NA | RT | PUB | Sanger + MLPA | rs121912664 | c.1010G>A | p.( Arg337His) |
| 181 / F | 3 | ACC | NA | RT | PUB | Sanger + MLPA | rs121912664 | c.1010G>A | p.( Arg337His) |
| 182 / F | 3 | ACC | NA | RT | PUB | NGS + MLPA | rs121912664 | c.1010G>A | p.( Arg337His) |
| 183 / F | 5 | ACC | NA | RT | PED | Sanger + MLPA | rs121912664 | c.1010G>A | p.( Arg337His) |
| 184 / F | 11 | ACC | NA | RT | PED | Sanger + MLPA | rs121912664 | c.1010G>A | p.( Arg337His) |
| 185 / M | 17 | ACC | NA | RT | PED | Sanger + MLPA | rs121912664 | c.1010G>A | p.( Arg337His) |
| 186 / F | 23 | Breast | NA | Familial, EOBC | PUB | Sanger + MLPA | rs121912664 | c.1010G>A | p.( Arg337His) |
| 187 / F | 57 | Breast | NA | Familial | PUB | Sanger + MLPA | rs121912664 | c.1010G>A | p.( Arg337His) |
| 188 / F | 49 | Breast (bilateral) | NA | Familial | PUB | Sanger + MLPA | rs121912664 | c.1010G>A | p.( Arg337His) |
| 189 / M | 1 | CNS (CPC) | NA | RT | PED | Sanger + MLPA | rs121912664 | c.1010G>A | p.( Arg337His) |
| 190 / M | 1 | CNS (CPC) | NA | RT | PED | Sanger + MLPA | rs121912664 | c.1010G>A | p.( Arg337His) |
| 191 / F | 1 | ACC | NA | Familial, RT | PED | Sanger + MLPA | rs121912664 | c.1010G>A | p.( Arg337His)* |

ACC, Adrenocortical Carcinoma; CNS, Central Nervous System; CPC, Choroid Plexus Carcinoma; EOBC, Early Onset Breast Cancer; MGPT, Multigene Panel Testing; MT, Multiple Tumors; MO, months old; OS, Osteosarcoma; RT, Rare Tumors, STS, Soft tissue sarcoma; NA, not applicable; PUB, high-risk public clinic; PC, high-risk private clinic; PED, pediatric tumors database; NGS, Next-generation Sequencing; MLPA, Multiplex Ligation-Dependent Probe Amplification; WT, wild-type genotype; * homozygous for the R337H variant.
