# Supplementary material for: Clinical and molecular characterization of patients fulfilling Chompret criteria for Li-Fraumeni syndrome in Southern Brazil
Source: PLoS One. 2021 Sep 16;16(9):e0251639. doi: 10.1371/journal.pone.0251639 (PMC8445435; doi:10.1371/journal.pone.0251639)
Supplement: Attachment — Submitted filename: Response to Reviewers 38705.docx [file pone.0251639.s004.docx]

Below please find a point-by-point response to the academic editor and reviewers’ comments:

**Academic Editor’s comments:**

After careful consideration, we feel that it has merit but does not fully meet PLOS ONE’s publication criteria as it currently stands. Therefore, we invite you to submit a revised version of the manuscript that addresses the points raised during the review process.

1. Include a CONSORT diagram to describe participant inclusion and exclusion. Provide clarity on whether all participants were from unique families or if there were any related cases. See additional comments from reviewers on descriptions of inclusion/exclusion.

**Reply:** We thank the editor for this suggestion. We have now included a consort diagram (Supplemental Figure S1) and highlighted that the participants were from unique families.

2. Consider adding in a new table to describe the overall cohort of the 191 study participants in a more concise manner with percentages etc. See both reviewer's comments.

**Reply:** We have presented a revised, more concise table as suggested by reviewers and the complete table will be provided as supplemental material.

3. Replace mutation with pathogenic variant per recently revised genetics terminology.

**Reply:** We thank the editor for this important suggestion. We have now corrected and standardized this terminology throughout the article text.

4. Provide more description on the technology used for pathogenic variant detection. If multiple methods were used were there differences in types of variants detected or rate of variant detection?

**Reply:** We have provided a diagram summarizing the different methods used for the patients included in the study. All methods allowed detection of both sequence variants and rearrangements. We did not observe drastic differences in variants detected with each method although we were unable to perform a formal statistical analysis since for some methods the number of patients analysed was small. The use of multiple testing methods was included in the discussion as a potential limitation of the study.

5. Update the Discussion per the reviewer's suggestions.

**Reply:** We have updated the discussion section to include reviewer's recommendations.

6. Consider adding in the suggested references.

**Reply:** We have included the suggested references.

7. Address both reviewer's comments related to putting this study into the context of the literature.

**Reply:** We thank the editor and reviewers for this comment and have added a sentence in the introduction to reinforce the context of the literature and contributions of our manuscript in this context.

**Journal Requirements:**

When submitting your revision, we need you to address these additional requirements.

1. Please ensure that your manuscript meets PLOS ONE's style requirements, including those for file naming. The PLOS ONE style templates can be found at

<https://journals.plos.org/plosone/s/file?id=wjVg/PLOSOne_formatting_sample_main_body.pdf>

and

<https://journals.plos.org/plosone/s/file?id=ba62/PLOSOne_formatting_sample_title_authors_affiliations.pdf>

**Reply:** We thank the editor for this important commentary. We have now modified the formatting of the manuscript to meet the style requirements according to the aforementioned templates.

2. Please provide additional details regarding participant consent. In the ethics statement in the Methods and online submission information, please ensure that you have specified:

- whether consent was informed

- when oral consent was obtained, how was it documented and witnessed

- whether parents or guardians signed the consent form for minors

**Reply:** We thank the editor for this commentary. We have now included the information about the consent.

3. Please provide the names of the cancer evaluation clinics.

**Reply:** The clinics were private consultation offices of the physicians and did not have a specific name, but we have included the names of the Hospital and main clinics where the private offices are situated in the consort diagram.

4. Please include captions for your Supporting Information files at the end of your manuscript, and update any in-text citations to match accordingly. Please see our Supporting Information guidelines for more information: <http://journals.plos.org/plosone/s/supporting-information>.

[Note: HTML markup is below. Please do not edit.]

**Reply: We thank the editor, and now have included the captions for our supporting information files.**

**Reviewers' comments:**

Reviewer's Responses to Questions

**1. Is the manuscript technically sound, and do the data support the conclusions?**

The manuscript must describe a technically sound piece of scientific research with data that supports the conclusions. Experiments must have been conducted rigorously, with appropriate controls, replication, and sample sizes. The conclusions must be drawn appropriately based on the data presented.

Reviewer #1: Yes

Reviewer #2: Yes

**2. Has the statistical analysis been performed appropriately and rigorously?**

Reviewer #1: I Don't Know

Reviewer #2: Yes

**3. Have the authors made all data underlying the findings in their manuscript fully available?**

The PLOS Data policy requires authors to make all data underlying the findings described in their manuscript fully available without restriction, with rare exception (please refer to the Data Availability Statement in the manuscript PDF file). The data should be provided as part of the manuscript or its supporting information, or deposited to a public repository. For example, in addition to summary statistics, the data points behind means, medians and variance measures should be available. If there are restrictions on publicly sharing data—e.g. participant privacy or use of data from a third party—those must be specified.

Reviewer #1: No

Reviewer #2: Yes

**4. Is the manuscript presented in an intelligible fashion and written in standard English?**

PLOS ONE does not copyedit accepted manuscripts, so the language in submitted articles must be clear, correct, and unambiguous. Any typographical or grammatical errors should be corrected at revision, so please note any specific errors here.

Reviewer #1: Yes

Reviewer #2: Yes

**5. Review Comments to the Author**

Please use the space provided to explain your answers to the questions above. You may also include additional comments for the author, including concerns about dual publication, research ethics, or publication ethics. (Please upload your review as an attachment if it exceeds 20,000 characters)

**Reviewer #1:**

This is an interesting manuscript which continues to add to our dynamic understanding of germline TP53 PVs. The manuscript would be aided by the following additions/changes:

-Please replace use of the term mutation in all places as this is no longer the preferred terminology.

-There are some notable omissions from the reference list that would be best cited including the following by LFS experts (David Malkin/ Judy Garber):

—>In particular during the discussion of the oligomerization domain: https://www.ncbi.nlm.nih.gov/pmc/articles/PMC6292786/

Lines 68-74: Reference work by Rana et al.

https://www.ncbi.nlm.nih.gov/pmc/articles/PMC6292786/

<https://pubmed.ncbi.nlm.nih.gov/31105275/>

-On variable penetrance of TP53 and meeting of Chompret criteria: You can contrast your findings in the discussion as there are notable differences likely based in the ascertainment of the cohorts studied, but it is worth contrasting these results.

-Why were some patients excluded? This is not explained. Please include a CONSORT diagram on how the 191 were arrived at.

-Please limit Table 1 to the TP53 pathogenic and likely pathogenic (PV) carriers only. The negative ones can be in a supplemental table.

Table 2: Instead of referring to mutations as ‘classic’ can you label them as DBD. Use of ‘classic’ in this context and in the table is confusing.

Relative risks or Odds ratios with 95% confidence intervals should be provided.

Table 3: For cohorts a/b: include median age at testing with IQR; include median age at first cancer diagnosis also with IQR.

Line 214 — comparison to literature should be in the discussion and not in the results section

Line 243 —include discussion of ascertainment

Line 250 — poor prognosis for what? Overall survival?

My understanding is that R337H was first identified due to the unusually high frequency of ACC, there is no mention of this well-established association or prior literature supporting the role of this variant in ACC. For example, https://pubmed.ncbi.nlm.nih.gov/15952083/ from 2005.

The discussion would be aided by referencing the findings of Pinto et al., which may help to explain potential modifiers of TP53 R337H : https://pubmed.ncbi.nlm.nih.gov/32637605/ from 2020.

**Reply:** We thank the reviewer 1 for the important suggestions. We have now included the suggested references and the consort diagram with the suggested informations which provides additional information on patient ascertainment. The issue of ascertainment was also included in the discussion. The table was changed to show the PV and LPV, we have now used the terminology DBD instead of "classic", updated the statistical analysis and information as suggested. Suggestions regarding Lines 214 and 250 suggestion were addressed.

**Reviewer #2:**

A good study examining the frequency of TP53 pathogenic variants (PV) in patients with cancer meeting the revised 2015 Chrompret criteria in Southern Brazil. The investigation of 191 cases builds the case for differences between age of onset and prevalence of cancer type between group A (classic mutations in the DNA binding domain of TP53) and group B (oligomerization domain encompassing the prevalent R337H PV in Brazil). Findings show a PV detection rate of 13.6% and that ACC is more prevalent in group B with a younger age of onset then other previous studies have suggested.

Comment one:

The abstract mentions “191 cancer affected and unrelated probands” (line 36) does unrelated probands mean all index cancer cases were independent and not from the same family? If so consider re-wording to “191 independent cancer cases from unique families”. The introduction would be aided with inclusion of literature on the prevalence of TP53 cases identified from the expansion of multi-gene panel testing (MGPT) in clinical practice and how this relates to targeting TP53 testing to cases that meet classic TP53 testing criteria and broadening of testing criteria.

**Reply:** We thank the reviewer and state that we have included the suggestion and changes in our manuscript.

Comment two:

In the methods section, it is not clear whether MGPT, Sanger sequencing or research testing using next generation sequencing (NGS) was applied retrospectively or just data collected retrospectively on a consecutive case series. This point needs clarifying to understand the origins of the data. If from retrospective data or application of testing retrospectively this needs to be mentioned in the limitations of the study also.

The methods section should include a statement in the first paragraph that all cancer cases and family histories meet revised 2015 Chrompret criteria and were selected for on this basis.

**Reply:** We thank the reviewer, we have updated our manuscript and believe that we now have a more clear information about the criterias.

Comment three:

The results section would be aided with a concise summary description of the population characteristics. Consider the expansive Table 1 as a supplementary table and instead summarise this data in a concise table with percentages for cancer type, gender, average age or age range of diagnosis, which aspect of chrompret criteria was meet, testing strategy and result. Suggest to organise case characteristics by negative and positive result or by group A or B. In this re-organisation it would be useful to understand the percentage of TP53 cases picked up by testing regimes MGPT, sanger or research NGS. This would link back to the inclusion of literature in introduction of TP53 pick up from MGPT in general. The existing supplementary table 1 defining the revised 2015 Chrompret criteria could be used alongside the above data to re-organise or link to expansive table 1 when moved to supplementary material. This would help the reader to digest and interpret the results better.

**Reply:** We thank the reviewer for this comment. We have presented a revised, more concise table as suggested by reviewers

Comment four:

For the reader the discussion would be aided by stating the main findings of the study from the start and then discuss each main finding in separate paragraphs. Inclusion of the emerging screening modalities for TP53 carriers ie MRI screening in adult TP53 carriers and paediatric screening protocol with attention to efficacy and benefit in the context of Brazil’s current funding model of genetic testing access and screening limits. There isn’t a comprehensive limitations section – need to include additional information on limitations ie study design in the nature of methods and statistical tools used and link to the future research direction information ie the use of different study designs in the application of MGPT with population control and cases included and other avenues of investigation to confirm the higher prevalence of R337H in group B and ACC with different ethnicities and larger data sets.

**Reply:** We thank the reviewer for this comment. We have improved our discussion including comments regarding limitations of the present study. We respectfully question inclusion of a more detailed description about the emerging cancer screening modalities for TP53 PV carriers since we believe that this is beyond the scope of the present study and for this reason we have not included changes regarding this issue.

We hope to have answered all points raised by the academic editor and reviewers and are ready to provide additional information as needed.

Kind regards,

**Patricia Ashton-Prolla, MD, PhD.**

**Genomic Medicine Laboratory, Experimental Research Center / Medical Genetics Service, Hospital de Clinicas de Porto Alegre**

**Ramiro Barcelos, 2350**

**90035-903 Porto Alegre-RS, Brazil**

**Tel + 55 51 3359-8011 / Fax + 55 51 3359-8010 /**
